# Digital Rights and Mobile Health in Low- and Middle-Income Countries: Protocol for a Scoping Review

**DOI:** 10.2196/49150

**Published:** 2023-10-03

**Authors:** Adam Poulsen, Yun J C Song, Eduard Fosch-Villaronga, Haley M LaMonica, Olivia Iannelli, Mafruha Alam, Ian B Hickie

**Affiliations:** 1 Brain and Mind Centre The University of Sydney Sydney Australia; 2 eLaw Center for Law and Digital Technologies Leiden University Leiden Netherlands

**Keywords:** human right, digital right, mobile health, mHealth, smartphone, mobile phone, digital health, scoping review, health equity, patient empowerment

## Abstract

**Background:**

Digital technology is a means to uphold or violate human rights in various domains, including business, military, and health. Given the pervasiveness of mobile technology in low- and middle-income countries (LMICs), mobile health (mHealth) interventions present an opportunity to reach remote populations and enable them to exercise civil and political rights and economic, social, and cultural rights, such as the right to health and education. Simultaneously, the ubiquity of mobile phones involves processing sensitive data which can threaten rights, including the right to privacy and nondiscrimination. Digital health is often promoted as advancing human rights and health equity; however, digital rights are underexplored in the literature on mHealth in LMICs. As such, creating an understanding of the digital rights topics covered in the 2022 literature is important to avoid exacerbating existing inequities relating to digital health design, use, implementation, and access.

**Objective:**

This scoping review aims to identify digital rights topics in the 2022 peer-reviewed literature on mHealth in LMICs.

**Methods:**

The Arksey and O’Malley framework for scoping reviews guides this review. Searches were performed across 7 electronic databases (Web of Science, Scopus, Ovid, ACM Digital Library, IEEE Xplore, ProQuest, and PubMed). The screening processes were guided by the research question “What digital rights topics have been explored in the 2022 literature on mHealth in LMICs?” Only papers addressing mHealth in LMICs and digital rights topics were included. Data extraction will include publication title, year, and type; first author’s affiliation country; LMICs implicated; infrastructure challenges; study aims, design, limitations, and future work; health area; mHealth technology, functions, purpose or application, and target end users; human or digital right terms used; explicit rights topics cited; and implied rights topics. The results will be reported using the PRISMA-ScR (Preferred Reporting Items for Systematic Reviews and Meta-Analyses Extension for Scoping Reviews) checklist.

**Results:**

This scoping review was registered in Open Science Framework (December 22, 2022). Title and abstract screening and full-text paper screening were completed in 2023. This resulted in 56 papers being included in the study. The target date for completing data extraction and publishing a case study of the initial findings is the end of 2023. The full scoping review findings are expected to be disseminated through various pathways benefiting academia, practice, and policy making by the end of 2024. These include journal papers, conference presentations, publicly available toolkits for research and practice, public webinars, and policy briefs with evidence-based policy recommendations emerging from this review.

**Conclusions:**

The planned scoping review will identify digital rights topics in the 2022 literature at the intersection of mHealth and LMICs. Furthermore, it will highlight the importance of patient empowerment, data protection, and inclusion in mHealth research and related policies in LMICs.

**Trial Registration:**

Open Science Framework osf.io/7mz24; https://osf.io/7mz24

**International Registered Report Identifier (IRRID):**

DERR1-10.2196/49150

## Introduction

### Background

In 2022, the European Commission signed “The Declaration on Digital Rights and Principles,” acknowledging the power of digital technologies as enablers and inhibitors of human rights [1]. Further, it reshapes and applies existing human rights to the digital space (eg, the right to the protection of their personal data on the internet and the right to freedom of expression in the internet-based environment). Likewise, in 2020, the United Nations put forward the “Roadmap for Digital Cooperation,” which acknowledged that digital technology, as a tool, is a means to exercise human rights and violate them [2,3]. Similarly, “the resolution on the promotion, protection, and enjoyment of human rights on the internet” in 2016 [4] affirmed the importance of protecting and realizing human rights on the internet and other information and communications technology. Clearly, human rights relating to the digital environment, or digital rights, are an emerging and evolving topic that concerns 2 key areas. First, the extension, realization, and protection of existing human rights in the digital space and over personal, digitized data (eg, freedom of expression). Second, the discussion around emerging human rights to access, use, and participate in the digital space (eg, the right to internet access) [5]. For this study, digital rights are all existing and emerging human rights that extend to, and relate to accessing and protecting, participation in the digital space.

The internet as an enabling technology is often at the center of the discussion around digital rights, with arguments made for the right to internet access [6-8]. Although the European Commission and United Nations do not explicitly identify internet access as a right, they proclaim necessary internet access protections for the sake of safeguarding, or at least not intentionally stifling, human rights [1,2,9]. So far, several countries have recognized the right to internet access, including Greece [10] and India [11]. Yet, the status of this right is still being discussed at the international level [12]. Beyond internet access considerations, the topic of digital rights also concerns the broader information and communications technology environment and addresses, for instance, health care systems and respect for human dignity [13], automation and the right to work [14], and surveillance technologies and the right to freedom of movement [15].

### Low- and Middle-Income Countries

Digital rights discourse often focuses on high-income countries in which, in most cases, users enjoy significant digital access and considerable rights and legal protections [16]. This work seeks to explore low- and middle-income countries (LMICs) instead, which are more at risk of digital rights violations (eg, state-sanctioned internet shutdowns and internet-based censorship) [17-19] and where user rights are inadequately regulated [20,21].

### Mobile Health

This study examines the underexplored digital rights concerns relating to mobile health (mHealth) in LMICs. mHealth refers to “the use of mobile and wireless technologies to support the achievement of health objectives” [22], categorizing various devices such as mobile phones, wearable and implantable devices, tablet computers, and personal digital assistants [23]. Digital health, including mHealth, is often promoted as advancing human rights and promoting “health equity, if implemented in partnership with communities and based on core values of local autonomy, fairness and ecological sustainability” [24]. However, to what extent the literature on mHealth in LMICs engages with digital rights topics is currently underexplored. Exemplary digital rights topics concerning mHealth in LMICs include the impacts of mandatory Subscriber Identity Module registration mobile app download taxes, cyberviolence over mobile phones, and internet-based censorship, as well as gender disparities and the gender digital divide, internet-based surveillance and the right to privacy, and prolonged data retention and the right to be forgotten [25-28]. To create knowledge in this underexplored space, this scoping review aims to identify digital rights topics in the 2022 peer-reviewed literature on mHealth in LMICs.

## Methods

### Scoping Review

This review follows the scoping review framework outlined by Arksey and O’Malley [29] and recommendations by Levac et al [30]. This involves adhering to and reporting the following six steps: (1) identifying the research question; (2) identifying relevant studies; (3) study selection; (4) charting the data; (5) collating, summarizing, and reporting results; and (6) consultation [29]. The PRISMA-ScR (Preferred Reporting Items for Systematic Reviews and Meta-Analyses Extension for Scoping Reviews) checklist will be used to report the review results. This review commenced in November 2022 and is expected to conclude by the end of 2023.

### Step 1: Identifying the Research Question

The guiding question of this review is “What digital rights topics have been explored in the 2022 literature on mHealth in LMICs?” To limit the scope to digital rights, this review will not investigate well-explored mobile access topics (eg, infrastructure, literacy, connectivity, and cost) except in instances relating to digital rights. Further, the scope is limited to literature published in 2022 based on resource limitations and the saliency and engagement of digital rights topics in recent years.

### Step 2: Identifying Relevant Studies

Searches were performed across 7 electronic databases. A preliminary search was performed on November 25, 2022, across 6 electronic databases: Web of Science, Scopus, Ovid, ACM Digital Library, IEEE Xplore, and ProQuest. The search strategy (Multimedia Appendix 1) consisted of terms considered to capture the nature of the research question. Multimedia Appendix 1 reports the numerous variations and abbreviations of terms used, as well as the search string adapted according to the requirements of each database. A second series of searches were performed on March 9, 2023, in the same databases to identify any papers published after the initial search date. On August 16, 2023, PubMed was added to the list of databases and searched. Search results were imported into Covidence (Veritas Health Innovation), a web-based tool for collaborative paper screening, reviewing, and data collection. Duplicate citations were removed using Covidence functions.

### Step 3: Study Selection

The inclusion and exclusion criteria applied to the study selection process are described in Textbox 1.

**Inclusion and exclusion criteria.**

**Inclusion criteria**

- Paper type: Peer-reviewed papers.
- Language: English.
- Publication date: 2022.
- Subjects of interest: Mobile health (mHealth) in low- and middle-income countries (LMICs) and digital rights topics.

**Exclusion criteria**

- Paper type: Not peer-reviewed papers (eg, unpublished work and gray literature).
- Language: All other languages.
- Publication date: Before or after 2022.
- Subjects of interest: Not mHealth in LMICs (eg, reviews on health technology in LMICs not covering mHealth) and not digital rights topics specifically (eg, mobile phone accessibility topics such as cost and digital literacy).

Papers were screened by 2 reviewers (coauthors AP and YJCS) to assess relevance to the research question over two iterations: (1) title and abstract screening and (2) full-text paper screening. To aid in both screening iterations, the authors developed a screening form and integrated it into Covidence. Following recommendations by Levac et al [30], the reviewers regularly met during the screening process to resolve emerging conflicts related to study selection. An arbiter (coauthor EFV) settled the conflicts that could not be resolved in the reviewers’ discussions.

Initially, during the search and title abstract screening, no limits were put on the publication date. However, the criteria were revised post hoc only to include publications from 2022 based on resource limitations and familiarity with the literature. As such, the search and title and abstract screening included papers published on any date, and only after the title and abstract screening were papers published before 2022 excluded. Post hoc, iterative revisions to the eligibility criteria are not uncommon [29,31] and are appropriate “to ensure that abstracts selected are relevant for full paper review” [30]. However, they should be reported with justification for transparency [32], as done here. The post hoc excluded papers will be retained for a later, larger review.

### Step 4: Charting the Data

For the included papers, 2 authors (AP and YJCS) independently piloted the data extraction process with 5 papers to reach a consensus and check for consistency. Afterward, data extraction of the remaining papers will be conducted by one reviewer (AP). Data will be extracted using a data chartering form integrated into Covidence, initially developed by the authors before the data collection process and later revised by 3 coauthors (AP, YJCS, and HML) after the data extraction pilot. Data items included in the form were publication title, year, and type; first author’s affiliation country; LMICs implicated; infrastructure challenges noted; study aims, design, limitations, and future work proposed; health area addressed through mHealth; mHealth technology, functions, purpose or application, and target end users; human or digital right terms used; explicit digital or human rights topics cited; and implied digital or human rights topics.

### Step 5: Collating, Summarizing, and Reporting Results

All data will be exported from Covidence and collated in a single spreadsheet on Excel (Microsoft Corp) for frequency and content analysis. Descriptive statistics will be calculated to summarize the general characteristics of the included literature. Content analysis will be used to interpret the extracted data. These methods are typical and recommended in scoping reviews [30].

### Step 6: Consultation

No consultation outside of the primary author’s wider research team will be performed in the design of this scoping review.

### Ethical Considerations

As this scoping review will not include human or animal participants, neither ethics approval nor participant consent is required. Only secondary analyses will be conducted on data collected from published literature.

## Results

**Figure 1:**
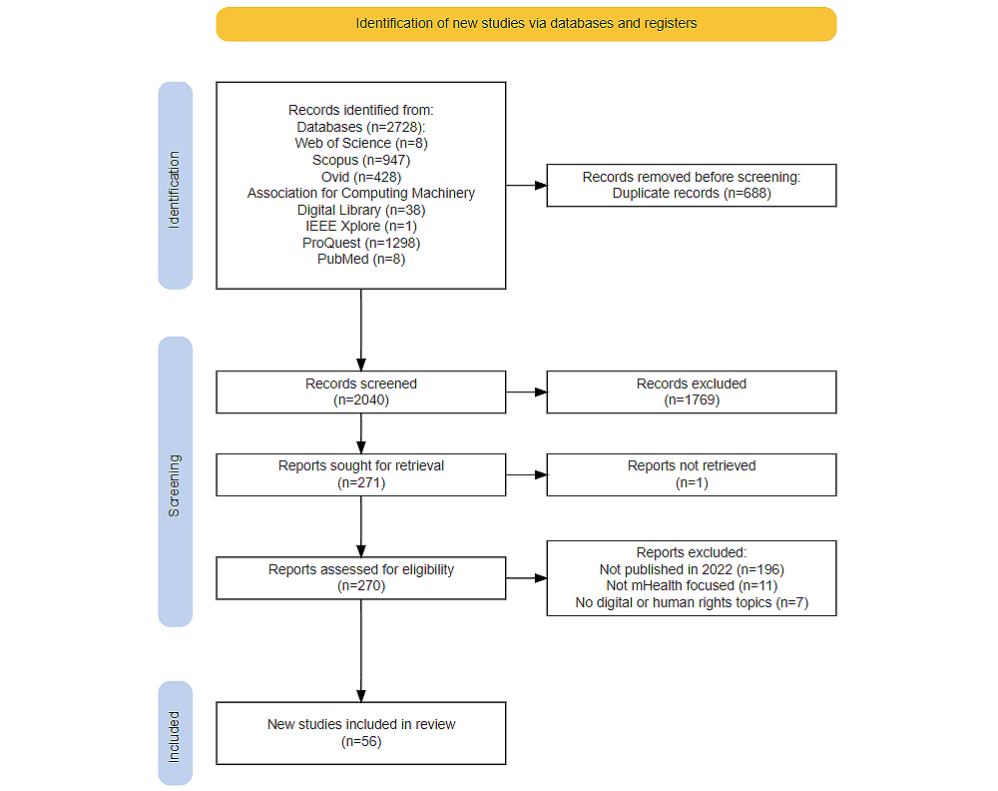
PRISMA-ScR (Preferred Reporting Items for Systematic Reviews and Meta-Analyses Extension for Scoping Reviews) flow diagram of the scoping review process.

The database searches yielded 2728 papers. After duplicates were removed, 2040 papers underwent title and abstract screening. This resulted in 271 papers being considered eligible for full-text screening. However, only 270 were retrieved for full-text screening (one was inaccessible and therefore excluded). Post hoc eligibility criteria were applied before full-text screening, excluding 196 not published in 2022. The post hoc excluded papers will be retained for a larger review to be completed in the future. The remaining 74 papers were considered eligible for inclusion. Following full-text paper screening of 74 papers, which excluded papers not focusing on mHealth (n=11) and not addressing digital or human rights topics (n=7), 56 papers were included in this scoping review. Figure 1 reports the scoping review process using the PRISMA-ScR flow diagram.

Data extraction is underway and expected to be completed by the end of 2023. Further, by the end of 2023, the initial findings of selected studies are expected to be published as a case study. The full scoping review findings are expected to be published by the end of 2024.

## Discussion

### Principal Results

This planned scoping review is designed to identify and map digital rights topics explored in the recent literature addressing mHealth in LMICs. Furthermore, it is projected to examine to what extent the literature engages with digital rights considerations relating to mHealth interventions; that is, whether digital rights topics are explicitly or implicitly deliberated. The review findings are expected to draw attention to the importance of data protection, inclusion, and patient empowerment in mHealth solutions and associated policies in LMICs to avoid exacerbating existing inequities relating to digital health design, use, implementation, and access. Moreover, this review is positioned to reveal existing knowledge and policy gaps relating to digital rights, which could help inform future mHealth research directions and relevant policy. The dissemination of findings will be varied, including journal papers, conference presentations, and publicly available research toolkits. Policymakers will be engaged through, for instance, webinars and policy briefs with evidence-based policy recommendations.

Due to the ubiquity of mobile phones in LMICs and continued interest in mHealth research in these contexts, this review will likely interest digital health researchers, making them aware of digital rights considerations when realizing and investigating digital health interventions. Furthermore, the findings of this review may benefit policy makers in LMICs by adding to the emerging discussion about digital rights protections and violations in these settings, which may impact digital health access.

### Limitations

This scoping review will follow well-established methods established by Arksey and O’Malley [29] and Levac et al [30]; yet, limitations will exist. First, scoping reviews lack formal bias and quality assessment methods, risking potential bias in the selection of included papers and derived conclusions [33]. Second, only peer-reviewed, English-language papers will be included in the review, thus excluding possible insights from the gray and non–English-language literature. As such, further research is needed to evaluate the quality of existing literature in this space and widen the scope of the included literature. Yet, acknowledging the strength of scoping reviews in providing an initial assessment of the size and scope of the available research literature, this review will provide the groundwork for research addressing digital rights and mHealth in LMICs going forward.

### Conclusions

This scoping review will identify digital rights topics in the 2022 peer-reviewed literature on mHealth in LMICs. Moreover, it will highlight the importance of data protection, patient empowerment, and inclusion in mHealth research and related policies in LMICs. In addition to creating knowledge about the digital rights topics currently deliberated or implied in the recent mHealth research literature, the review findings will identify to what extent researchers engage with those topics. Furthermore, the results will identify knowledge and policy gaps and future research directions relevant to mHealth in LMICs research. The findings may advantage digital health researchers and policymakers operating in LMIC contexts, improving understanding of pertinent digital rights considerations relating to mHealth.

## Appendix

Multimedia Appendix 1 Search strategy.

## Abbreviations

LMIC: low- and middle-income country
mHealth: mobile health
PRISMA-ScR: Preferred Reporting Items for Systematic Reviews and Meta-Analyses Extension for Scoping Reviews

## Data Availability

The data sets generated and analyzed during this study will be made available from the corresponding author upon reasonable request.
