# Supplementary material for: Digital Rights and Mobile Health in Low- and Middle-Income Countries: Protocol for a Scoping Review
Source: JMIR Res Protoc. 2023 Oct 3;12:e49150. doi: 10.2196/49150 (PMC10582819; doi:10.2196/49150)
Supplement: Multimedia Appendix 1 [file resprot_v12i1e49150_app1.docx]

## Search strategy, by database

### Web of Science Core Collection

ALL=("mobile health" OR "mHealth") AND ALL=("digital right*" OR "digital human right*" OR "human right*" OR "fundamental right*" OR "universal right*" OR "natural right*" OR "unalienable right*" OR "freedom of" OR "freedom to" OR "right to") AND ALL=("low- and middle-income countr*" OR "lower- and middle-income countr*" OR "low-income countr*" OR "middle-income countr*" OR "low-middle income countr*" OR "lower-middle income countr*" OR "low/middle-income countr*" OR "lower/middle-income countr*" OR "low and lower middle income countr*" OR "LMIC" OR "developing countr*" OR "developing world")

### Scopus

ALL("mobile health" OR "mHealth") AND ALL("digital right*" OR "digital human right*" OR "human right*" OR "fundamental right*" OR "universal right*" OR "natural right*" OR "unalienable right*" OR "freedom of" OR "freedom to" OR "right to") AND ALL("low- and middle-income countr*" OR "lower- and middle-income countr*" OR "low-income countr*" OR "middle-income countr*" OR "low-middle income countr*" OR "lower-middle income countr*" OR "low/middle-income countr*" OR "lower/middle-income countr*" OR "low and lower middle income countr*" OR "LMIC" OR "developing countr*" OR "developing world")

### Ovid (All Databases)

("mobile health" or "mHealth") and ("digital right*" or "digital human right*" or "human right*" or "fundamental right*" or "universal right*" or "natural right*" or "unalienable right*" or "freedom of" or "freedom to" or "right to") and ("low- and middle-income countr*" or "lower- and middle-income countr*" or "low-income countr*" or "middle-income countr*" or "low-middle income countr*" or "lower-middle income countr*" or "low/middle-income countr*" or "lower/middle-income countr*" or "low and lower middle income countr*" or "LMIC" or "developing countr*" or "developing world")

### The ACM Guide to Computing Literature

("mobile health" OR "mHealth") AND ("digital right*" OR "digital human right*" OR "human right*" OR "fundamental right*" OR "universal right*" OR "natural right*" OR "unalienable right*" OR "freedom of" OR "freedom to" OR "right to") AND ("low- and middle-income countr*" OR "lower- and middle-income countr*" OR "low-income countr*" OR "middle-income countr*" OR "low-middle income countr*" OR "lower-middle income countr*" OR "low/middle-income countr*" OR "lower/middle-income countr*" OR "low and lower middle income countr*" OR "LMIC" OR "developing countr*" OR "developing world")

### IEEE Xplore (Mandatory Limitation to 8 Wildcards)

("mobile health" OR "mHealth") AND ("digital right" OR "digital rights" OR "digital human right" OR "digital human rights" OR "human right" OR "human rights" OR "fundamental right" OR "fundamental rights" OR "universal right" OR "universal rights" OR "natural right" OR "natural rights" OR "unalienable right" OR "unalienable rights" OR "freedom of" OR "freedom to" OR "right to") AND ("low- and middle-income countr*" OR "lower- and middle-income countr*" OR "low-income countr*" OR "middle-income countr*" OR "low-middle income countr*" OR "lower-middle income countr*" OR "low/middle-income countr*" OR "lower/middle-income countr*" OR "low and lower middle income country" OR "low and lower middle income countries" OR "LMIC" OR "developing country" OR "developing countries" OR "developing world")

### ProQuest

[STRICT] ("mobile health" OR "mHealth") AND ("digital right*" OR "digital human right*" OR "human right*" OR "fundamental right*" OR "universal right*" OR "natural right*" OR "unalienable right*" OR "freedom of" OR "freedom to" OR "right to") AND ("low- and middle-income countr*" OR "lower- and middle-income countr*" OR "low-income countr*" OR "middle-income countr*" OR "low-middle income countr*" OR "lower-middle income countr*" OR "low/middle-income countr*" OR "lower/middle-income countr*" OR "low and lower middle income countr*" OR "LMIC" OR "developing countr*" OR "developing world")

### PubMed

(("mobile health") OR ("mHealth")) AND (("digital right*") OR ("digital human right*") OR ("human right*") OR ("fundamental right*") OR ("universal right*") OR ("natural right*") OR ("unalienable right*") OR ("freedom of") OR ("freedom to") OR ("right to")) AND (("low- and middle-income countr*") OR ("lower- and middle-income countr*") OR ("low-income countr*") OR ("middle-income countr*") OR ("low-middle income countr*") OR ("lower-middle income countr*") OR ("low/middle-income countr*") OR ("lower/middle-income countr*") OR ("low and lower middle income countr*") OR ("LMIC") OR ("developing countr*") OR ("developing world"))
